# Ex Vivo Expansion of Human CD8^+^ T Cells Using Autologous CD4^+^ T Cell Help

**DOI:** 10.1371/journal.pone.0030229

**Published:** 2012-01-12

**Authors:** Marcus O. Butler, Osamu Imataki, Yoshihiro Yamashita, Makito Tanaka, Sascha Ansén, Alla Berezovskaya, Genita Metzler, Matthew I. Milstein, Mary M. Mooney, Andrew P. Murray, Hiroyuki Mano, Lee M. Nadler, Naoto Hirano

**Affiliations:** 1 Department of Medical Oncology, Dana-Farber Cancer Institute, Massachusetts, United States of America; 2 Department of Medicine, Brigham and Women's Hospital, Massachusetts, United States of America; 3 Department of Medicine, Harvard Medical School, Boston, Massachusetts, United States of America; 4 Division of Functional Genomics, Jichi Medical University, Tochigi, Japan; 5 Department of Medical Genomics, University of Tokyo, Tokyo, Japan; 6 Immune Therapy Program, Campbell Family Institute for Breast Cancer Research, Campbell Family Cancer Research, Ontario Cancer Institute, Toronto, Ontario, Canada; 7 Department of Immunology, University of Toronto, Toronto, Ontario, Canada; New York University, United States of America

## Abstract

**Background:**

Using *in vivo* mouse models, the mechanisms of CD4^+^ T cell help have been intensively investigated. However, a mechanistic analysis of human CD4^+^ T cell help is largely lacking. Our goal was to elucidate the mechanisms of human CD4^+^ T cell help of CD8^+^ T cell proliferation using a novel *in vitro* model.

**Methods/Principal Findings:**

We developed a genetically engineered novel human cell-based artificial APC, aAPC/mOKT3, which expresses a membranous form of the anti-CD3 monoclonal antibody OKT3 as well as other immune accessory molecules. Without requiring the addition of allogeneic feeder cells, aAPC/mOKT3 enabled the expansion of both peripheral and tumor-infiltrating T cells, regardless of HLA-restriction. Stimulation with aAPC/mOKT3 did not expand Foxp3^+^ regulatory T cells, and expanded tumor infiltrating lymphocytes predominantly secreted Th1-type cytokines, interferon-γ and IL-2. In this aAPC-based system, the presence of autologous CD4^+^ T cells was associated with significantly improved CD8^+^ T cell expansion *in vitro*. The CD4^+^ T cell derived cytokines IL-2 and IL-21 were necessary but not sufficient for this effect. However, CD4^+^ T cell help of CD8^+^ T cell proliferation was partially recapitulated by both adding IL-2/IL-21 and by upregulation of IL-21 receptor on CD8^+^ T cells.

**Conclusions:**

We have developed an *in vitro* model that advances our understanding of the immunobiology of human CD4^+^ T cell help of CD8^+^ T cells. Our data suggests that human CD4^+^ T cell help can be leveraged to expand CD8^+^ T cells *in vitro*.

## Introduction

It is now well accepted that neoplastic cells are immunogenic and that tumors develop in the context of immune recognition by the host [1], [2]. Tumor-associated antigens that serve as immune targets include cell lineage differentiation antigens, cancer-testes antigens, and neoantigens produced by mutations in the cancer cell's unstable genome. Mutational events can give rise to multiple immunogenic MHC class I and II restricted, non-self epitopes capable of inducing strong immune responses to the tumor [3], [4]. In several maligancies, anti-tumor T cell responses, with infiltration of tumors by CD8^+^ T lymphocytes and local production of interferon-γ and IL-2, have been associated with improved clinical prognosis [5]–[8].

Counter regulatory immune responses, however, also develop in the cancer-bearing host. Tumors subvert the immune response by secreting chemotactic factors that recruit immune suppressive elements, thereby inhibiting the function of anti-tumor effectors [9]. Tumor infiltration by T regulatory (Treg) cells has been correlated with inferior clinical outcomes in several tumors [10], [11]. These findings have led to the proposal that immune recognition of cancer involves the balancing of opposing forces: anti-tumor effectors vs. pro-tumor regulatory elements [10], [12], [13]. In fact, a high ratio of Treg cells to CD8^+^ T cells within the tumor microenvironment has been associated with poorer survival [14], [15].

Adoptive T cell therapy is a promising treatment modality designed to amplify the anti-tumor immune response. Anti-tumor effectors are expanded *in vitro*, away from the pro-tumor milieu of the cancer bearing host, and then reinfused as a cellular therapy [16]–[21]. Successful approaches showing clinical activity include adoptive transfer of tumor antigen-specific T cell lines or clones that have been derived from the peripheral blood. Specificity can be achieved by stimulating antigen-specific precursor T cells or through genetic modification of expanded bulk T cells to express cloned or chimeric T cell receptor (TCR) genes [22]–[26]. Alternatively, the nascent, endogenous immune effector response to the tumor can be amplified by expanding tumor-infiltrating lymphocytes (TIL) *in vitro*. Adoptive cell transfer of *in vitro* activated TIL has achieved major clinical responses when patients first undergo lymphodepletion and are then given high dose IL-2 after adoptive transfer [17], [27]. Lymphodepletion augments the persistence and function of transferred TIL not only by reducing or temporarily eliminating Treg cells, but also by reducing cytokine sinks that results in the accumulation of homeostatic cytokines such as IL-7 and IL-15 [28], [29].

The optimal method for generating clinically effective T cell grafts *in vitro* has yet to be established [21], [30]. In order to achieve massive numerical expansion of T cells, current methods necessitate the use of soluble monoclonal antibodies (mAb), allogeneic feeder PBMC, EBV transformed lymphoblastoid cell lines, and/or undefined culture supernatants. Consequently, these requirements present formidable challenges and costs that prevent the widespread clinical application of this therapy. While adoptive transfer of anti-tumor CD4^+^ T cells can be efficacious, expansion of anti-tumor CD8^+^ T cells is also an important goal, particularly in light of the association between their persistence and clinical responses [18], [31]–[33].

Insights into requirements for augmenting the expansion of both CD4^+^ and CD8^+^ T cells will help further improve methods to generate T cell grafts for adoptive therapy. CD4^+^ T cells help generate effective immune responses by sustaining CD8^+^ T cell proliferation, preventing exhaustion, and establishing long-lived functional memory [34]. In mouse models, common γ-chain receptor cytokine and CD40 signaling can mediate CD4^+^ T cell help [34]–[44]. In clinical studies, CD4^+^ T cells have also been implicated in promoting the persistence and anti-tumor activity of antigen-specific CD8^+^ T cells in patients [45], [46]. However, the mechanisms of human CD4^+^ T cell help are less well understood. To conduct a mechanistic analysis of human CD4^+^ T cell help, we developed a novel, human cell-based aAPC, aAPC/mOKT3, which induces both CD4^+^ and CD8^+^ T cell expansion without allogeneic feeder cells. The removal of allogeneic feeder cells from our T cell culture system enabled us to precisely isolate molecules mediating help of CD8^+^ T cell expansion that are expressed or secreted by human CD4^+^ T cells.

## Results

### K562-based aAPC expressing membranous OKT3 induces CD3^+^ T cell expansion

**Figure 1:**
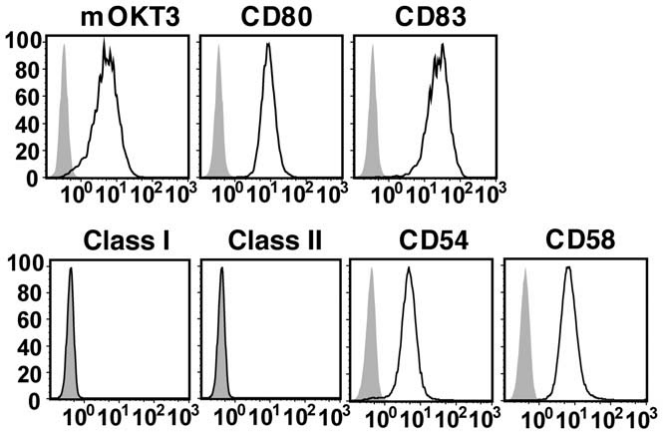
Generation of aAPC/mOKT3. Surface expression of a transduced membranous form of anti-CD3 mAb, and transduced CD80, CD83, and endogenous HLA class I, class II, CD54, and CD58 on aAPC/mOKT3 is shown. A membranous form of anti-CD3 mAb on aAPC/mOKT3 (open) and wild type K562 (shaded) was stained using goat anti-mouse IgG (H+L). Other surface molecules were stained with each specific mAb (open) and isotype control (shaded) and analyzed by flow cytometry. Note the lack of endogenous expression of HLA class I and II on aAPC/mOKT3.

We and others have previously reported the generation of aAPC derived from the human erythroleukemia cell line K562 [47]–[51]. K562 serves as an excellent platform for generating aAPC since it expresses no HLA class I or II molecules, but highly expresses adhesion molecules such as CD54 and CD58. Using K562, we developed a novel aAPC, aAPC/mOKT3, capable of expanding CD3^+^ T cells regardless of HLA subtype (Figure 1A, Figure S1). This aAPC was engineered to express a membranous form of the anti-CD3 mAb, OKT3, on its cell surface, thus obviating the need for adding soluble mAb to T cell cultures or loading it onto aAPC as described elsewhere [51], [52]. aAPC/mOKT3 also ectopically expresses immunostimulatory molecules CD80 and CD83. We and others have shown that CD83 delivers a CD80 dependent signal that promotes lymphocyte longevity [47], [53], [54].

### Stimulation of CD3^+^ T cells with aAPC/mOKT3 induces robust CD8^+^ T cell expansion

**Figure 2:**
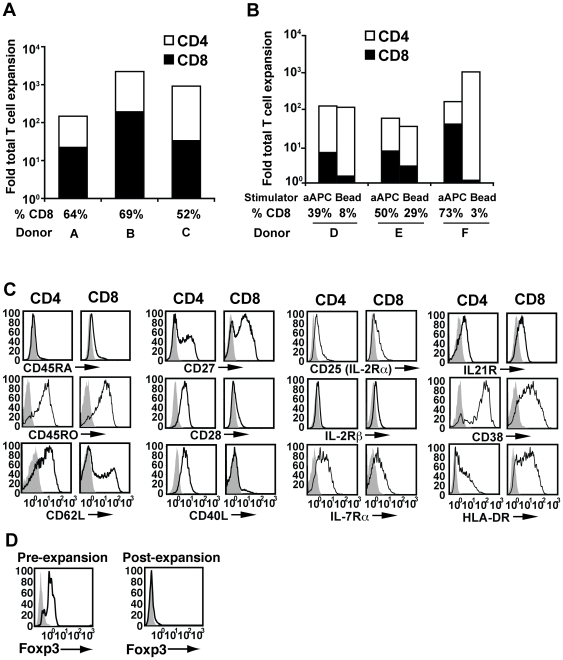
aAPC/mOKT3 expands both CD4^+^ and CD8^+^ T cells without using allogeneic feeder PBMC. (**A**) CD3^+^ T cells were stimulated twice with aAPC/mOKT3 and supplemented with IL-2 between stimulations. Fold expansion of CD3^+^ T cells over one month is shown for three donors. Shading shows the proportion of expanded CD4^+^ (white) and CD8^+^ (black) T cells, and percent CD8^+^ T cells is indicated. (**B**) CD3^+^ T cells were stimulated twice with aAPC/mOKT3 or beads (Dynabeads CD3/CD28) and supplemented with IL-2 between stimulations. Fold expansion of CD3^+^ T cells over one month is shown for three donors. Shading shows the proportion of expanded CD4^+^ (white) and CD8^+^ (black) T cells, and percent CD8^+^ T cells is indicated. (**C**) CD3^+^ T cells were expanded as described in Figure 2A. Expression of surface molecules on gated CD4^+^ and CD8^+^ T cells is shown (open). Isotype mAb staining was used as a control (shaded). (**D**) CD4^+^ CD25^+^ Foxp3^+^ Treg cells, present pre-expansion, were absent in expanded cultures. CD4^+^ CD25^+^ cells, pre- and post-expansion, were stained intracellularly with anti-Foxp3 mAb (open) and isotype control (shaded).

Peripheral CD3^+^ T cells expanded with aAPC/mOKT3 were phenotypically characterized after 28 days in culture (Figure 2). While the number of both CD4^+^ and CD8^+^ T cells increased, CD8^+^ T cells expanded substantially better than CD4^+^ T cells, and therefore dominated cultures from every donor tested (Figure 2A). This is in contrast to other pan T cell expansion systems such as anti-CD3/CD28 mAb-coated beads, which invariably favor the expansion CD4^+^ T cells over CD8^+^ T cells [55] (Figure 2B). Similar fold expansion of CD3^+^ T cells was obtained with the aAPC/mOKT3-based and antibody-coated bead-based expansion systems. T cells expanded using aAPC/mOKT3 displayed a central memory∼effector memory phenotype (CD45RA^-^ CD54RO^+^ CD62L^+/-^) and retained expression of receptors for IL-2, IL-7, and IL-21 (Figure 2C). CD40 ligand was highly expressed by CD4^+^ T cells but not CD8^+^ T cells. Importantly, expanded CD4^+^ CD25^+^ T cells did not express Foxp3, indicating that immunoinhibitory Treg cells did not proliferate well (Figure 2D).

### aAPC/mOKT3 induces unbiased CD3^+^ T cell expansion, preserving the repertoire for viral and tumor-associated antigens

**Figure 3:**
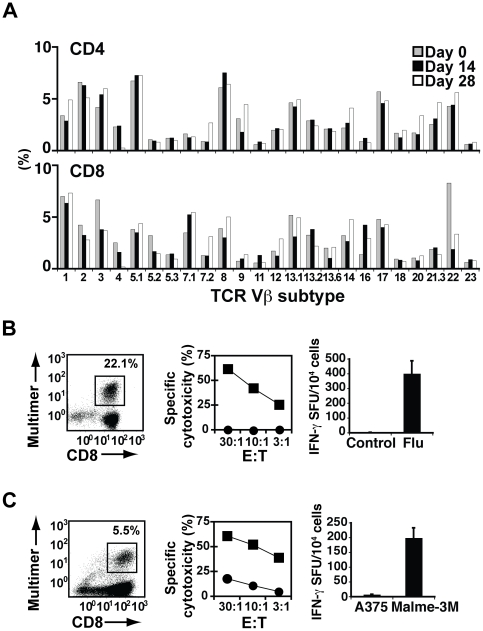
Expansion with aAPC/mOKT3 does not induce skewing of the TCR Vβ repertoire. (**A**) TCR Vβ subfamily analysis before and after stimulation with aAPC/mOKT3 is shown. CD3^+^ T cells were stimulated with aAPC/mOKT3 on days 0 and 14 and were treated with IL-2 at 300 IU/ml between stimulations. TCR Vβ usage analysis was performed on days 0, 14, 28. Data shown is on gated CD4^+^ and CD8^+^ T cells. (**B, C**) A2^+^ CD3^+^ T cells were stimulated twice with aAPC/mOKT3 for one month. Subsequently, CD8^+^ T cells were purified from expanded CD3^+^ T cells and further stimulated with aAPC/A2 pulsed with Flu or MART1 peptide. (**B**) Flu specificity was demonstrated by multimer staining (left). Functional competence was demonstrated by antigen-specific cytotoxicity (middle) and IFN-γ secretion (right). T2 cells pulsed with Flu peptide (▪) or control peptide (•) were used as targets. (**C**) MART1 specificity was similarly demonstrated by multimer staining (left). The HLA-A2^+^/MART1^+^ melanoma line, Malme-3M (▪), and the HLA-A2^+^/MART1^-^ melanoma line, A375 (•), were used as targets in cytotoxicity (middle) and IFN-γ ELISPOT assays (right).

In order to evaluate whether stimulation with aAPC/mOKT3 induced broad expansion of CD3^+^ T cells, TCR Vβ repertoire analysis was performed. No obvious skewing in the TCR Vβ usage of both CD4^+^ and CD8^+^ T cell populations was revealed, supporting “unbiased” T cell expansion by aAPC/mOKT3 (Figure 3A). Moreover, HLA-restricted antigen-specific CD8^+^ cytotoxic T lymphocytes (CTL) against viral and tumor antigens could be generated from CD3^+^ T cells initially expanded for four weeks using aAPC/mOKT3 (Figure 3B and 3C). The functional avidity of these tumor antigen-specific T cells was sufficient to recognize tumor targets endogenously expressing antigen, confirming that the T cell repertoire for tumor antigen recognition was preserved (Figure 3C). We also confirmed that stimulation with aAPC/mOKT3 induced the expansion of tumor-antigen specific T cells. After 28 days in culture, MART1 peptide specific CD8^+^ T cell expansion was 420–1,150 fold (Figure S1D).

### aAPC/mOKT3 expands functional TIL but not contaminating Treg cells

**Figure 4:**
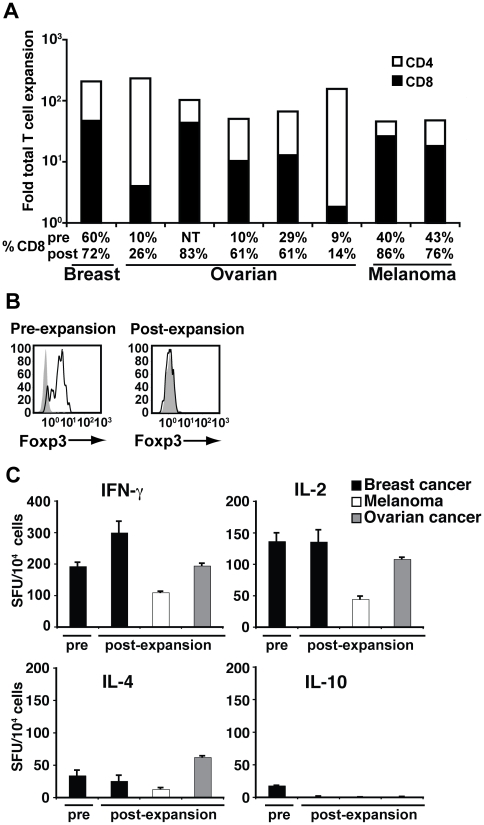
aAPC/mOKT3 expanded TIL are Foxp3 negative and secrete predominantly Th1 cytokines. (**A**) Expansion of TIL obtained from breast and ovarian cancer ascites and melanoma metastases is shown. Shading indicates the proportion of CD4^+^ (white) and CD8^+^ (black) T cells in expanded cultures. The percentage of CD8^+^ T cells in pre- and post-expansion cultures is shown. Note that in all samples tested, the percentage of CD8^+^ T cells increased even in those that initially contained a minimal percentage of CD8^+^ T cells. NT denotes not tested. (**B**) CD4^+^ CD25^+^ Foxp3^+^ Treg cells, present pre-expansion, were not detectable after one month of culture. CD4^+^ CD25^+^ cells were intracellularly stained with anti-Foxp3 mAb (open) and isotype control (shaded). (**C**) IFN-γ, IL-2, IL-4, and IL-10 secretion of expanded TIL was determined by ELISPOT assays. Cytokine secretion by TIL from the breast cancer ascites specimen prior to expansion is shown as a control. Pre-expansion samples from melanoma and ovarian cancer specimens were not studied because of low initial cell numbers.

Using aAPC/mOKT3, lymphocytes derived from malignant ascites (breast and ovarian cancer) and melanoma metastases were successfully expanded without adding any allogeneic feeder cells (Figure 4A). As observed with peripheral CD3^+^ T cells in Figure 2A, CD8^+^ T cells predominantly expanded in all cultures, including those that initially contained a minimal percentage of CD8^+^ T cells. Importantly, Foxp3^+^ cells did not proliferate well (Figure 4B). As with peripheral CD3^+^ T cells, expanded TIL had a central memory∼effector memory phenotype (CD45RA^-^ CD62L^+/-^) consistent with a lack of terminal differentiation (Figure S2). Furthermore, expanded T cells highly expressed CD27 and CD28 which are associated with T cell survival and persistence *in vivo* [56]-[59]. They also secreted high quantities of IFN-γ and IL-2, while IL-4 secretion was lower and no IL-10 was produced (Figure 4C). These results demonstrate that the aAPC/mOKT3-based system can expand tumor-infiltrating CD8^+^ T cells in the presence of autologous CD4^+^ T cells, and that they display phenotypic and functional characteristics consistent with central memory∼effector memory T cells.

### IL-2 and IL-21 are necessary, but not sufficient, for CD4^+^ T cell-mediated help of CD8^+^ T cell expansion

**Figure 5:**
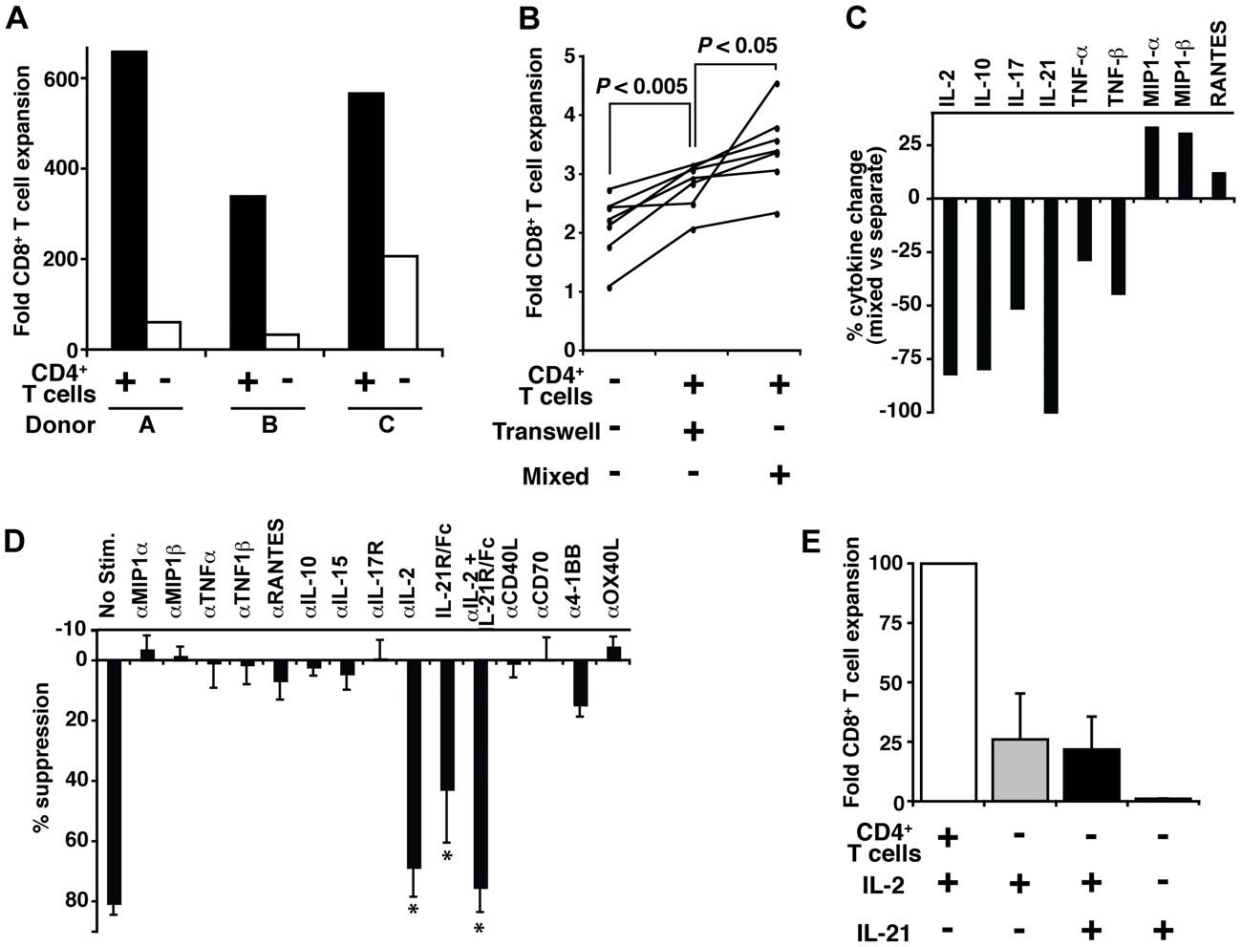
Autologous CD4^+^ T cell secretion of IL-2/IL-21 is necessary but not sufficient to help CD8^+^ T cells proliferate. (**A**) CD8^+^ T cells were stimulated twice by aAPC/mOKT3 with or without CD4^+^ T cells and treated with IL-2 between stimulations. Fold expansion of CD8^+^ T cells over 28 days is shown for 3 donors. (**B**) CD8^+^ T cells were stimulated only once by aAPC/mOKT3 with or without CD4^+^ T cells in transwell plates. No IL-2 or other cytokines were given. Fold expansion of CD8^+^ T cells over 6 days is shown for 7 donors. (**C**) Culture supernatants were tested for a panel of soluble factors to identify mediators of CD4^+^ T cell help. Relative changes in cytokines, comparing mixed vs. separate cultures, are shown. Data is representative of two donors. Absolute values for two donors are shown in Table S1. (**D**) Suppression of CD8^+^ T cell expansion in the presence of CD4^+^ T cells by blocking reagents is presented as percent suppression relative to control. Values indicate mean of four independent experiments; error bars show s.d. *P<0.005. (**E**) CD8^+^ T cells were stimulated twice with aAPC/mOKT3 in the presence or absence of CD4^+^ T cells. IL-2, IL-21, or both were added in each condition. Fold expansion of CD8^+^ T cells over 28 days is shown. Percent expansion was calculated by dividing the number of expanded CD8^+^ T cells by the number of CD8^+^ T cells expanded in the presence of CD4^+^ T cells. Values indicate mean of six independent experiments; error bars show s.d.

Using the aAPC/mOKT3-based expansion system, we compared the expansion of CD8^+^ T cells in the presence or absence of CD4^+^ T cells. CD8^+^ T cells expanded much better in the presence of CD4^+^ T cells (Figure 5A), suggesting the presence of CD4^+^ T cell help for CD8^+^ T cells in these aAPC/mOKT3-based cultures. We tested whether this “help” was mediated by soluble factors or cell-cell contact using the transwell assay (Figure 5B). A single stimulation, without any exogenously added cytokines, expanded CD8^+^ T cells by an average of 40.5% better when CD4^+^ T cells were present but separated from CD8^+^ T cells by the transwell membrane (*P*<0.005). In co-cultures where CD4^+^ and CD8^+^ T cells were mixed, allowing for direct cell-cell contact, CD8^+^ T cells expanded more than in cultures where they were separated from CD4^+^ T cells by the transwell membrane (*P*<0.05). These results suggest that observed CD4^+^ T cell help involves both soluble factors and cell-cell contact.

To identify molecules mediating the observed CD4^+^ T cell help, culture supernatants of CD4^+^/CD8^+^ T cell mixed and separate cultures were tested for a panel of soluble factors (Figure 5C and Table S1). Greater quantities of MIP-1α, MIP-1β, and RANTES were detected in CD4^+^/CD8^+^ T cell mixed cultures compared to separate cultures, suggesting increased production in mixed cultures. In contrast, IL-2 and IL-21, as well as IL-10, IL-17, TNF-α, and TNF-β, were detected at lower levels in mixed cultures, consistent with more consumption or less production of these cytokines.

To differentiate between “more consumption” and “less production,” CD4^+^/CD8^+^ T cell mixed cultures were stimulated in the presence of blocking reagents, and suppression of CD8^+^ T cell expansion was assessed (Figure 5D). Blockade of IL-2 and IL-21 resulted in a reduction of expansion by 68.8% (*P*<0.005) and 42.9% (*P*<0.005), respectively. These results indicate that the decreased levels of IL-2 and IL-21 in CD4^+^/CD8^+^ T cell mixed cultures were due to more consumption rather than less production and that these cytokines may be necessary mediators of CD4^+^ T cell help in this human-based in vitro system. To test whether IL-2/IL-21 could substitute for the observed CD4^+^ T cell help, CD8^+^ T cells stimulated with aAPC/mOKT3 were supplemented with IL-2, IL-21, or both (Figure 5E). CD8^+^ T cells did not expand without IL-2. The addition of IL-2 with or without IL-21 did not improve CD8^+^ T cell expansion to the level observed when cocultured with CD4^+^ T cells, demonstrating that IL-2 plus IL-21 are not sufficient to replace CD4^+^ T cell help.

### Exogenous IL-2/IL-21 and upregulation of IL-21 receptor can partially recapitulate CD4^+^ T cell help of CD8^+^ T cell expansion *in vitro*

**Figure 6:**
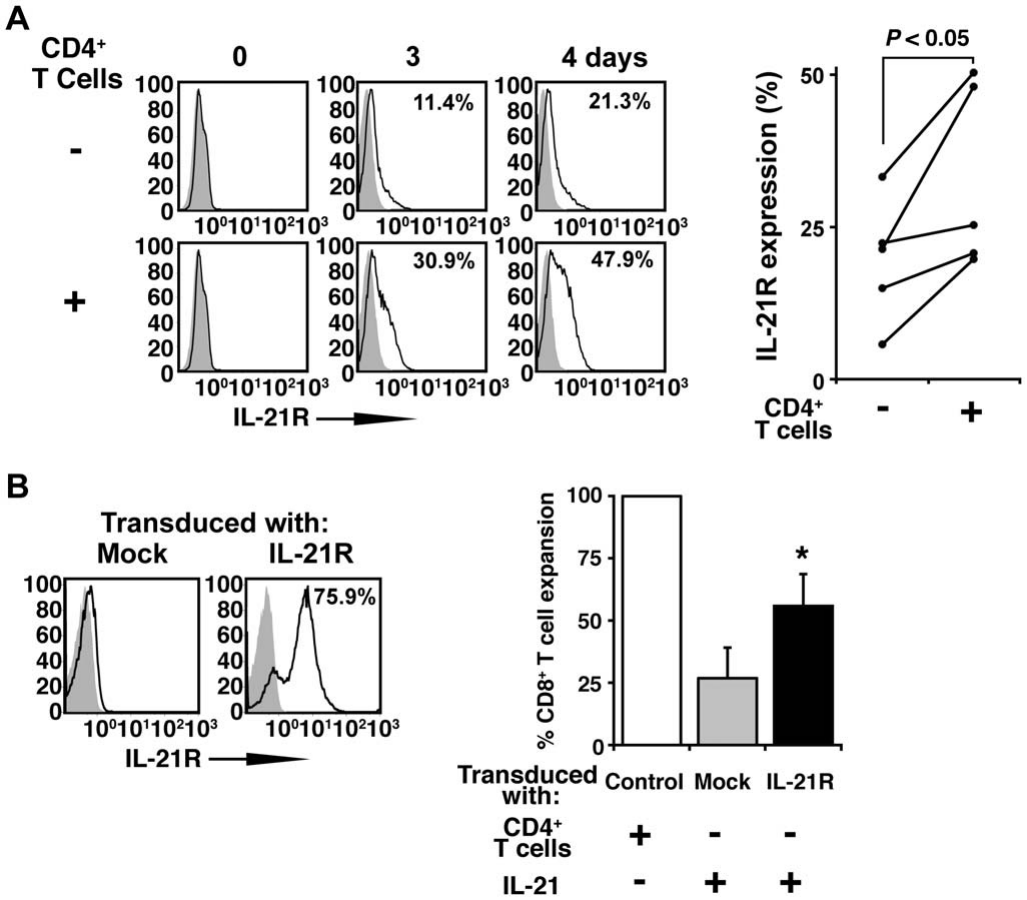
IL-2/IL-21 and upregulation of IL-21R expression replace CD4^+^ T cell help of CD8^+^ T cell expansion *in vitro.* (**A**) IL-21R expression on CD8^+^ T cells stimulated with aAPC/mOKT3 in the presence or absence of CD4^+^ T cells was studied by flow cytometry. On the left, histogram plots for 1 donor is shown and, on the right, IL-21R expression on day 4 is displayed for 5 donors. (**B**) IL-21R expression on CD8^+^ T cells ectopically transduced with mock or IL-21R is shown (left). Expansion of transduced CD8^+^ T cells stimulated twice by aAPC/mOKT3 with or without IL-21 is compared (right). Percent expansion was calculated by dividing the number of expanded transduced CD8^+^ T cells by that of CD8^+^ T cells stimulated in the presence of CD4^+^ T cells. Values indicate mean of four independent experiments; error bars show s.d. *P<0.005.

Interestingly, we observed that higher expression of the IL-21 receptor (IL-21R) on CD8^+^ T cells occurred when CD4^+^ T cells were present during stimulation by aAPC/mOKT3 (Figure 6A). Higher IL-21R expression on CD8^+^ T cells was not induced by supplementing cultures with IL-2 and IL-21 (data not shown). This prompted us to hypothesize that increased upregulation of IL-21R on CD8^+^ T cells is critical for the full effect of IL-21 secreted by CD4^+^ T cells. We constitutively expressed IL-21R on CD8^+^ T cells (Figure 6B, left) and stimulated them with aAPC/mOKT3 in the presence of IL-2/IL-21. In accordance with the transduction efficiency of IL-21R to 75.9%, CD8^+^ T cell proliferation partially increased to levels seen in the presence of CD4^+^ T cells (Figure 6B, right). This indicates that elevated expression of IL-21R is necessary and can partially recapitulate CD4^+^ T cell help for CD8^+^ T cell proliferation.

## Discussion

A novel human cell-based aAPC expanded CD3^+^ T cells *in vitro* without the addition of allogeneic feeder PBMC. Phenotypic analysis of expanded healthy donor T cells and TIL showed, that while both CD4^+^ and CD8^+^ T cells expanded, CD8^+^ T cells predominated. In this model system, we demonstrated that CD8^+^ T cell expansion depended on the presence of CD4^+^ T cells, suggesting that CD4^+^ T cells provided help to proliferating CD8^+^ T cells. The CD4^+^ T cell secreted cytokines, IL-2 and IL-21, and the CD4^+^ T cell-dependent upregulation of IL-21R on CD8^+^ T cells were necessary for the observed CD4^+^ T cell help.

IL-2 and IL-21 have previously been shown to mediate CD4^+^ T cell help in murine *in vivo* studies. IL-2, one of the few effector cytokines made by naïve CD4^+^ T cells, expands activated T cells and is essential in the development of CD8^+^ T cell memory responses to pathogens [60]. While CD8^+^ T cell responses during acute viral infections were relatively independent of IL-2, the development of protective CD8^+^ T cell memory responses required IL-2 exposure during priming [35]–[37]. In vivo models also indicate that IL-21 is critical for containing chronic viral infections and preventing the deletion of high affinity antiviral CD8^+^ T cells. IL-21 secretion by CD4^+^ T cells enables the generation, sustained proliferation, and maintenance of polyfunctional CD8^+^ T cells during chronic infection [39]–[41].

Our results confirmed a role for IL-2 and IL-21 in human CD4^+^ T cell help. By using a standardized aAPC, we were able to single out and examine the effects of cocultured CD4^+^ T cells, unhindered by immunostimulatory and inhibitory factors produced by allogeneic feeder cells. Stimulation of T cells with aAPC/mOKT3 induced the secretion of cytokines and chemokines, including high levels of interferon-γ, MIP-1α, and MIP-1β. Among all the cytokines and chemokines studied, blocking experiments identified IL-2 and IL-21 as necessary for CD4^+^ T cell help of CD8^+^ T cell expansion. These cytokines alone, however, were not sufficient to replace CD4^+^ T cells. We showed that CD4^+^ T cells help by enhancing IL-21R expression on CD8^+^ T cells, rendering them more responsive to secreted IL-21. Taken together, the secretion of IL-2/IL-21 and the induction of IL-21R are necessary and sufficient to partially recapitulate human CD4^+^ T cell help of CD8^+^ T cell expansion *in vitro*.

Transwell assays showed that the CD4^+^ T cell dependent expansion of CD8^+^ T cells was also mediated by cell-cell contact factors. CD40-CD40 ligand interactions have been shown to mediate CD4^+^ T cell help through CD40-mediated activation of dendritic cells, which are then “licensed” to stimulate CD8^+^ T cells [43], [44], [61]. CD40 ligation was also shown to increase IL-21R expression on B lymphocytes suggesting a mechanism for IL-21R upregulation on CD8^+^ T cells [62]. However, we did not observe any suppression of CD8^+^ T cell expansion following blockade of CD40 ligand (Figure 5D) even though expanded CD4^+^ T cells strongly expressed CD40 ligand (Figure 2C). Furthermore, stimulation with aAPC/mOKT3 in the presence of CD40 ligation and the addition of IL-21 did not consistently enhance CD8^+^ T cell expansion (data not shown). Therefore, these results are in agreement with others who have shown that CD4^+^ T cells do not provide direct help to CD8^+^ T cells through CD40 ligation [63], [64]. It should be noted that blocking of CD70, 4-1BB, or OX40 signaling also did not suppress the expansion of CD8^+^ T cells in the presence of CD4^+^ T cells (Figure 5D).

aAPC induced polyclonal expansion of both CD4^+^ and CD8^+^ T cells as shown by the absence of clonal skewing of the TCR Vβ repertoire. The ability to further expand antigen-specific T cells capable of killing tumor targets indicated that the TCR repertoire for highly avid T cells was preserved. Also, expanded TIL secreted higher amounts of Th1 cytokines, IFN-γ and IL-2, which are associated with anti-tumor immunity. While aAPC/mOKT3 induced substantial expansion of CD8^+^ T cells in the presence of CD4^+^ T cell help, terminal effector T cell differentiation did not occur, as demonstrated by the central memory∼effector memory phenotype (CD45RA^-^ CD45RO^+^ CD62L^+/-^). Retention of CD62L expression would enable homing to lymph nodes, where encounter with antigen presented by professional APC could augment immune responses [65]. CD27, which is down-regulated in late stage effector T cells, was also highly expressed. CD27 expression by *in vitro* expanded TIL and T cell clones has been associated with persistence and clinical responses after adoptive transfer [56], [57], [59], [66].

We also found that expanded T cells were not contaminated by cells with the CD4^+^ CD25^+^ Foxp3^+^ Treg phenotype even when CD4^+^ CD25^+^ Foxp3^+^ T cells were present prior to stimulation. We previously found that K562-based aAPC expressing HLA-DR molecules did not expand Foxp3^+^ cells even though aAPC itself produces modest amounts of the Treg cell growth factor TGF-β [48]. We previously reported that aAPC also secretes IL-6 [47]. It is possible that IL-6, secreted by aAPC, might interfere with Foxp3^+^ Treg cell expansion [67], [68].

Adoptive transfer of *in vitro* expanded T cells has led to clinically significant anti-tumor responses in patients [30]. By leveraging autologous CD4^+^ T cell help, aAPC/mOKT3 eliminates the use allogeneic feeder cells for T cell expansion, potentially increasing the availability of adoptive therapy as a cancer treatment. We previously reported the development of K562-based aAPCs dedicated to the expansion of HLA-restricted antigen-specific CD4^+^ and CD8^+^ T cells [47], [48]. Antigen-specific CD4^+^ and CD8^+^ T cells expanded *in vitro* with these aAPC had a central memory∼effector memory phenotype (CD45RA^-^ CD62L^+/-^) and possessed surprisingly prolonged *in vitro* longevity without feeder cells or cloning. In a recent clinical trial, HLA-A2-restricted MART1 peptide-specific CD8^+^ T cells generated *in vitro* with aAPC were infused to advanced melanoma patients [69]. Without lymphodepletion or IL-2 administration, transferred T cells could persist for >16 months, established anti-tumor immunological memory *in vivo*, trafficked to tumor, and induced clinical responses. aAPC/mOKT3 extends the K562 platform to the stimulation of T cells regardless of HLA subtype. The aAPC/mOKT3-based T cell expansion system facilitates the understanding of mechanisms for human CD4^+^ T cell help and provides a novel strategy to expand T cells for *in vitro* and *in vivo* uses.

## Materials and Methods

### Ethics Statement

All specimens and clinical data were collected under protocols approved by the Institutional Review Board at the Dana-Farber Cancer Institute (DFCI). All patients provided written informed consent for the collection of samples and subsequent analysis.

### cDNAs and cell lines

cDNAs encoding the heavy and light chains for a membranous form of anti-CD3 mAb (OKT3, mIgG2a) were cloned from hybridoma cells (ATCC, VA). HLA null K562 transduced with CD80 and CD83 has been described previously [47], [53]. CD80^+^ CD83^+^ K562 cells were retrovirally transduced with the heavy and light chains of a membranous form of anti-CD3 mAb. After drug selection, anti-CD3 mAb expressing cells were isolated by magnetic bead guided sorting (Miltenyi Biotec, CA). High expression of a membranous form of anti-CD3 mAb on the cell surface was confirmed by flow cytometry. The parental cell line K562 lacks the endogenous expression of any HLA molecule, but does endogenously express the adhesion molecules CD54 and CD58.

Retrovirus supernatants expressing IL-21R was harvested from PG13 cells. Fresh CD8^+^ T cells purified from healthy donors were first activated with anti-CD3 (0.75 µg/ml) and anti-CD28 (1 µg/ml) mAbs (Fitzgerald Industries International, MA) for two days. Pre-activated T cells were infected with IL-21R or mock retrovirus supernatants every 24 hr at an MOI of 10 for 10 days and treated with 50 IU/ml IL-2 between infections. Following the assessment of IL-21R expression by flow cytometry analysis, infected T cells were stimulated with aAPC/mOKT3.

T2, A375, and Malme-3M cell lines were obtained from ATCC as described elsewhere [47].

### T cell expansion

Healthy donor PBMC were obtained by leukapheresis performed at the DFCI Kraft Family Blood Donor Center. Cells were isolated by Ficoll-Hypaque density gradient centrifugation and CD3^+^, CD4^+^, or CD8^+^ T cells were purified by negative selection via MACS sorting according to the manufacturer's protocol (Miltenyi Biotec, CA). TIL samples were processed by centrifugation of malignant ascites or mechanical and enzymatic digestion of melanoma metastases with collagenase as previously described [70]. CD3^+^ TIL were obtained by positive or negative selection via MACS sorting (Miltenyi Biotec, CA). aAPC/mOKT3 cells were irradiated (200 Gy) and added to purified T cells at a T cell to aAPC ratio of 20∶1 unless otherwise noted. Dynabeads CD3/CD28 (Invitrogen, CA) were used as stimulators according to the manufacturer's instruction at a T cell to bead ratio of 1∶3. Expanding T cells were cultured in RPMI 1640 containing 10% human AB sera and gentamycin (Invitrogen, CA), and between stimulations, unless otherwise noted, 300 IU/ml IL-2 (Prometheus, CA) was added every 3-4 days. In the absence of CD4^+^ T cells, CD8^+^ T cells expanded only in the presence of IL-2. Where indicated, 50 ng/ml IL-21 (Peprotech, NJ) was added every 3-4 days. Unless otherwise noted, T cells were restimulated every two weeks. Expanded cells were characterized two weeks after the second stimulation. Cell viability was >90% by trypan blue exclusion.

To test whether antigen-specific cultures can be generated from CD3^+^ T cells polyclonally expanded with aAPC/mOKT3, CD3^+^ T cells derived from HLA-A*0201 (A2)^+^ donors were initially stimulated and expanded with aAPC/mOKT3 for one month. Subsequently, CD8^+^ T cells were purified and further stimulated with Flu or MART1 peptide-pulsed aAPC/A2 as previously described [47], [53].

### Analysis of cultured T cells

Flow cytometry analysis was performed using mAbs for the following antigens: CD4, CD8, CD25, CD28, CD56, CD62L, and IL-2Rβ (Coulter, CA); CD40 ligand, CD80, IL-7Rα, OX40, OX40 ligand, and 4-1BB (BD Biosciences, CA); CD27, CD45RA, CD45RO and CD83 (Invitrogen, CA); CCR4 and CCR7 (R&D Systems, MN); ICOS, NKG2D, and PD-1 (eBioscience, CA); CD38, Foxp3, HLA-DR, and 4-1BB ligand (Biolegend, CA); CD40 and CD70 (Ancell, MN); IL-21R (R&D Systems, MN; or BD Biosciences, CA). Goat anti-mouse IgG (H+L) Fab (Jackson ImmunoResearch, PA) was used to detect surface expression of murine Ig. Assessment of TCR Vβ subfamily usage was performed using TCR Vβ mAbs (Beta Mark, Coulter, CA).

To assess the production/consumption of soluble factors in T cell cultures, purified CD4^+^, CD8^+^, or a 1∶1 mixture of CD4^+^ and CD8^+^ T cells were stimulated with irradiated aAPC/mOKT3 for 72 hours and supernatants were measured for: GM-CSF, IFN-γ, IL-2, IL-4, IL-10, IL-12, IL-15, IL-17, MIP-1α, MIP-1β, RANTES, TNF-α, TNF-β, and TRAIL (R&D Systems, MN); IL-7 (Diaclone/Cell Sciences, MA); IL-18 (Medical & Biological Laboratories, Japan); and IFN-α (PBL Biomedical Laboratories, NJ). IL-21 (eBiosciences, CA) was measured at 48-hours. Relative changes in cytokines resulting from mixed cultures of CD4^+^ and CD8^+^ T cells vs. separate CD4^+^ and CD8^+^ T cell cultures were determined by the following formula: (x-y)/y, where x = cytokine secreted by CD4^+^ and CD8^+^ T cell mixed co-cultures and y is the average of cytokine produced in separately stimulated CD4^+^ and CD8^+^ T cell cultures.

IFN-γ ELISPOT and standard chromium release assays were performed as described elsewhere [47], [53]. IL-2, IL-4 and IL-10 ELISPOT assays were performed according to the manufacturer's protocol (R&D Systems, MN).

### Transwell and blocking assays

Transwell assays were performed by placing purified CD4^+^, CD8^+^, or a mixture of CD4^+^ and CD8^+^ T cells into Millicell-24 plate chambers (Millipore) which were separated by a 0.4 µm filter allowing free movement of soluble factors but not cells. T cells were stimulated once with aAPC/mOKT3 in the absence of exogenous cytokines. Six days later, expansion of CD8^+^ T cells was determined.

Blocking assays were performed in 96-well round bottomed plates where CD4^+^ and CD8^+^ T cells were combined 1∶1 and then stimulated with irradiated mOKT3/aAPC in the presence of blocking reagents. Blocking mAbs used recognized IL-2, IL-10, IL-15, IL-17R, MIP-1α, MIP-1β, OX40 ligand, RANTES, TNFα, and TNFβ (R&D Systems, MN); 4-1BB (Neomarkers, CA); CD40 ligand (Biolegend, CA); and CD70 (Ancell, MN). IL-21 was blocked using recombinant human IL-21R subunit/Fc chimeric protein (R&D Systems, MN) as previously described [71]. Six days later, CD8^+^ T cell expansion was determined.

### Statistical analysis

Data analysis was performed using the paired, one-sided Student's t-test where *P*<0.05 was considered to be statistically significant.

## Supporting Information

Figure S1 **K562-based aAPC/mOKT3, expressing a membranous form of anti-CD3 mAb, stimulates CD3^+^ T cell expansion. (A)** CD3^+^ T cells were stimulated twice with aAPC/mOKT3 and supplemented with IL-2 at the following concentrations: 10 IU/ml (gray), 300 IU/ml (white) and 6,000 IU/ml (black). Fold expansion over 28 days is demonstrated. Without IL-2 addition, T cell expansion over the 28-day culture period was minimal. Data for three separate donors is shown. **(B)** CD3^+^ T cells were stimulated twice with aAPC/mOKT3 at the indicated aAPC: T cell ratios. Cultures were supplemented with IL-2 (300 IU/ml) between stimulations. Fold expansion of CD3^+^ T cells over one month is shown for two donors. **(C)** Phenotype of fresh healthy donor CD3^+^ T cells prior to stimulation is depicted to compare with the T cells shown in Figure 2C which were expanded with aAPC/mOKT3. Expression of surface molecules on gated CD4^+^ and CD8^+^ T cells is shown (open). Isotype mAb staining was used as a control (shaded). **(D)** HLA-A2^+^ healthy donor CD8^+^ T cells were stimulated with MART1 peptide-pulsed aAPC/A2 as previously described [47], [53]. MART1 specific T cells were then stimulated twice with aAPC/mOKT3 in the presence of autologous CD4^+^ T cells. Fold expansion of MART1 T cells over one month is shown for three donors.
(TIF)

Figure S2 **TIL expanded with aAPC/mOKT3 express CD27 and CD28 and have a central memory∼effector memory phenotype.** CD3^+^ T cells from malignant ovarian ascites were stimulated twice with aAPC/mOKT3, and cultures were supplemented with IL-2 at 300 IU/ml. **(A)** Fresh, unstimulated TIL and **(B)** aAPC/mOKT3 expanded TIL were stained with indicated mAb (open) and isotype control (shaded). TIL were analyzed after a one month expansion. Data depicted is on gated CD4^+^ and CD8^+^ T cells.
(TIF)

Table S1 **Soluble factors in T cell cultures stimulated with aAPC/mOKT3.** Concentrations of soluble factors (pg/ml) in supernatants of CD4^+^ separate, or CD8^+^ separate, and CD4^+^ and CD8^+^ mixed T cell cultures stimulated by aAPC/mOKT3 were measured by ELISA. ^a^Percent change was calculated as detailed in Methods. ^b^not applicable. Data from two different donors is depicted.
(DOC)
