# Supplementary figures and images for: Ex Vivo Expansion of Human CD8+ T Cells Using Autologous CD4+ T Cell Help
Source: PLoS One. 2012 Jan 12;7(1):e30229. doi: 10.1371/journal.pone.0030229 (PMC3257268; doi:10.1371/journal.pone.0030229)

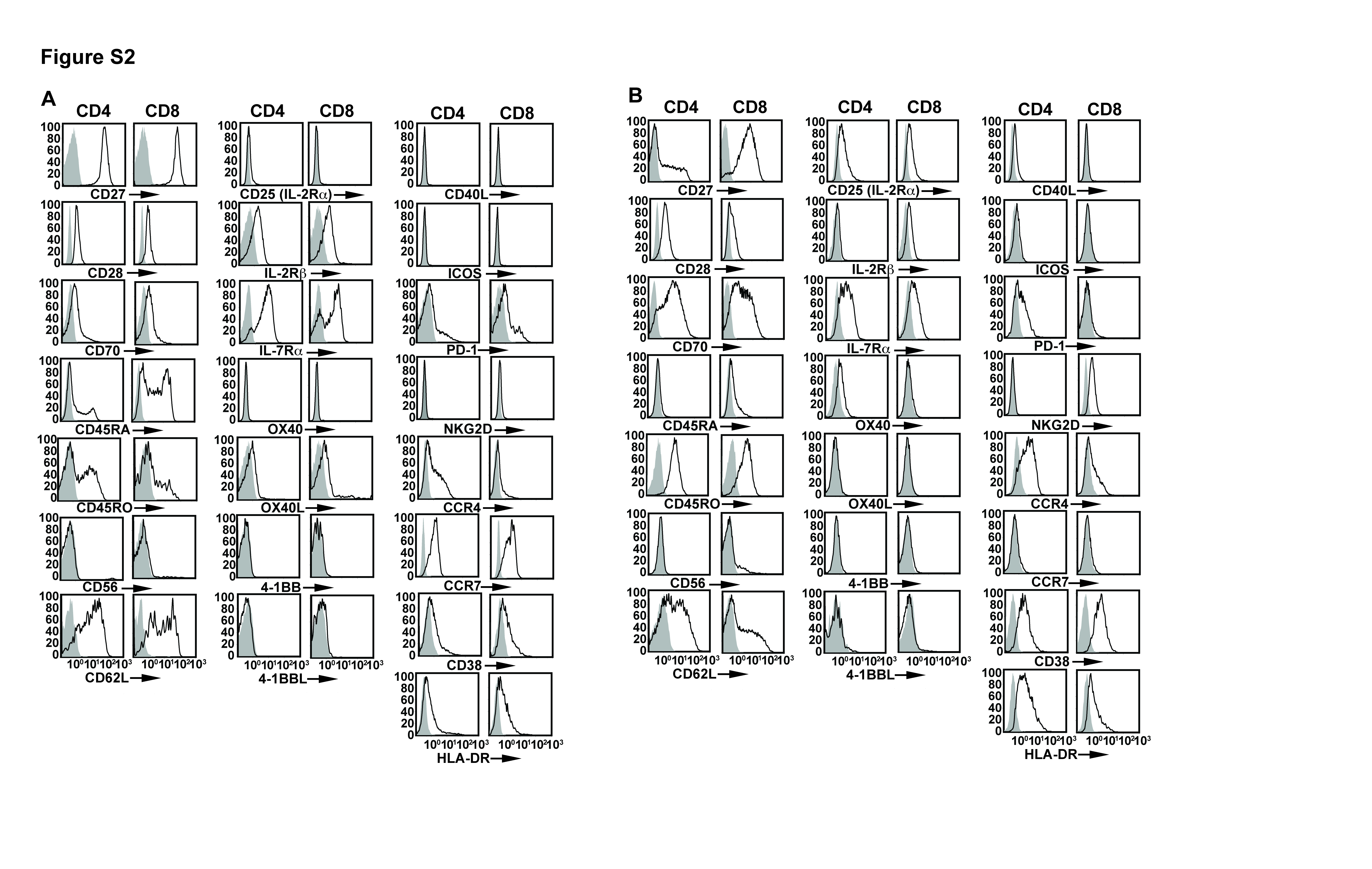

Supplement: Figure S2 — TIL expanded with aAPC/mOKT3 express CD27 and CD28 and have a central memory∼effector memory phenotype. CD3+ T cells from malignant ovarian ascites were stimulated twice with aAPC/mOKT3, and cultures were supplemented with IL-2 at 300 IU/ml. (A) Fresh, unstimulated TIL and (B) aAPC/mOKT3 expanded TIL were stained with indicated mAb (open) and isotype control (shaded). TIL were analyzed after a one month expansion. Data depicted is on gated CD4+ and CD8+ T cells. (TIF) [file pone.0030229.s002.tif]
