# Supplementary material for: Ex Vivo Expansion of Human CD8+ T Cells Using Autologous CD4+ T Cell Help
Source: PLoS One. 2012 Jan 12;7(1):e30229. doi: 10.1371/journal.pone.0030229 (PMC3257268; doi:10.1371/journal.pone.0030229)
Supplement: Table S1 — Soluble factors in T cell cultures stimulated with aAPC/mOKT3. Concentrations of soluble factors (pg/ml) in supernatants of CD4+ separate, or CD8+ separate, and CD4+ and CD8+ mixed T cell cultures stimulated by aAPC/mOKT3 were measured by ELISA. aPercent change was calculated as detailed in Methods. bnot applicable. Data from two different donors is depicted. (DOC) [file pone.0030229.s003.doc]

**Table S1. Soluble factors in T cell cultures stimulated with aAPC/mOKT3**

| **Cytokine**  **(pg/ml)** | **CD4^+^**  **separate** | **CD8^+^**  **separate** | **CD4^+^ and CD8^+^**  **mixed (1:1)** | **Percent change^a^**  **mixed vs. separate**  **cultures** |
| --- | --- | --- | --- | --- |
| **IL-2** | 3,720±13 | 1,180±4 | 1,163±10 | -52.5% |
|  | 1,722±13 | 111±0 | 164±6 | -82.1% |
| **IL-10** | 563±5 | 4±0 | 57±4 | -79.8% |
|  | 1,060±9 | 8±2 | 120±3 | -77.8% |
| **IL-17** | 985±12 | 12±0 | 242±2 | -51.4% |
|  | 1,149±31 | 14±3 | 338±2 | -41.9% |
| **IL-21** | 101±0 | < assay | < assay | -100% |
|  | 38±0 | < assay | < assay | -100% |
| **TNF-α** | 2,153±15 | 415±10 | 1,083±18 | -15.6% |
|  | 1,215±14 | 249±6 | 522±8 | -28.7% |
| **TNF-β** | 2,010±77 | 755±49 | 989±10 | -28.5% |
|  | 1,572±60 | 365±1 | 538±7 | -44.5% |
| **MIP-1α** | 19,785±461 | 5,719±107 | 14,257±330 | +11.8% |
|  | 7,917±201 | 4,343±79 | 8,217±556 | +34.0% |
| **MIP-1β** | 12,133±228 | 9,548±70 | 13,768±371 | +27.0% |
|  | 5,031±43 | 5,708±164 | 7,038±39 | +31.1% |
| **RANTES** | 3,544±50 | 1,793±51 | 3,006±2 | +12.7% |
|  | 1,864±1 | 1,521± 33 | 1,856±6 | +9.7% |
| **GM-CSF** | 4,708±169 | 4,242±173 | 4,891±476 | +9.3% |
|  | 3,913±53 | 2,166±170 | 2,999±2 | -1.3% |
| **IFN-γ** | 30,353±98 | 10,018±213 | 23,889±210 | +18.3% |
|  | 35,769±579 | 3,051±76 | 18,618±470 | -4.1% |
| **IL-18** | 18±0 | 17±0 | 19±1 | +5.9% |
|  | 20±1 | 17±0 | 18±1 | -4.4% |
| **IFN-α** | < assay | < assay | < assay | n/a^b^ |
|  | < assay | < assay | < assay | n/a |
| **IL-4** | < assay | < assay | < assay | n/a |
|  | < assay | < assay | < assay | n/a |
| **IL-7** | < assay | < assay | < assay | n/a |
|  | < assay | < assay | < assay | n/a |
| **IL-12** | < assay | < assay | < assay | n/a |
|  | < assay | < assay | < assay | n/a |
| **IL-15** | < assay | < assay | < assay | n/a |
|  | < assay | < assay | < assay | n/a |
| **TRAIL** | < assay | < assay | < assay | n/a |
|  | < assay | < assay | < assay | n/a |

Concentrations of soluble factors (pg/ml) in supernatants of CD4^+^ separate, or CD8^+^ separate, and CD4^+^ and CD8^+^ mixed T cell cultures stimulated by aAPC/mOKT3 were measured by ELISA. ^a^Percent change was calculated as detailed in Methods. ^b^not applicable. Data from two different donors is depicted.
